# Supplementary material for: The Gender-Biased Differential Effect of KDM6A Mutation on Immune Therapy in Urothelial Carcinoma: A Public Database Study
Source: Cancers (Basel). 2025 Jan 22;17(3):356. doi: 10.3390/cancers17030356 (PMC11816370; doi:10.3390/cancers17030356)
Supplement: Supplementary file 1 [file cancers-17-00356-s001.zip › cancers-3375849-supplementary.pdf]

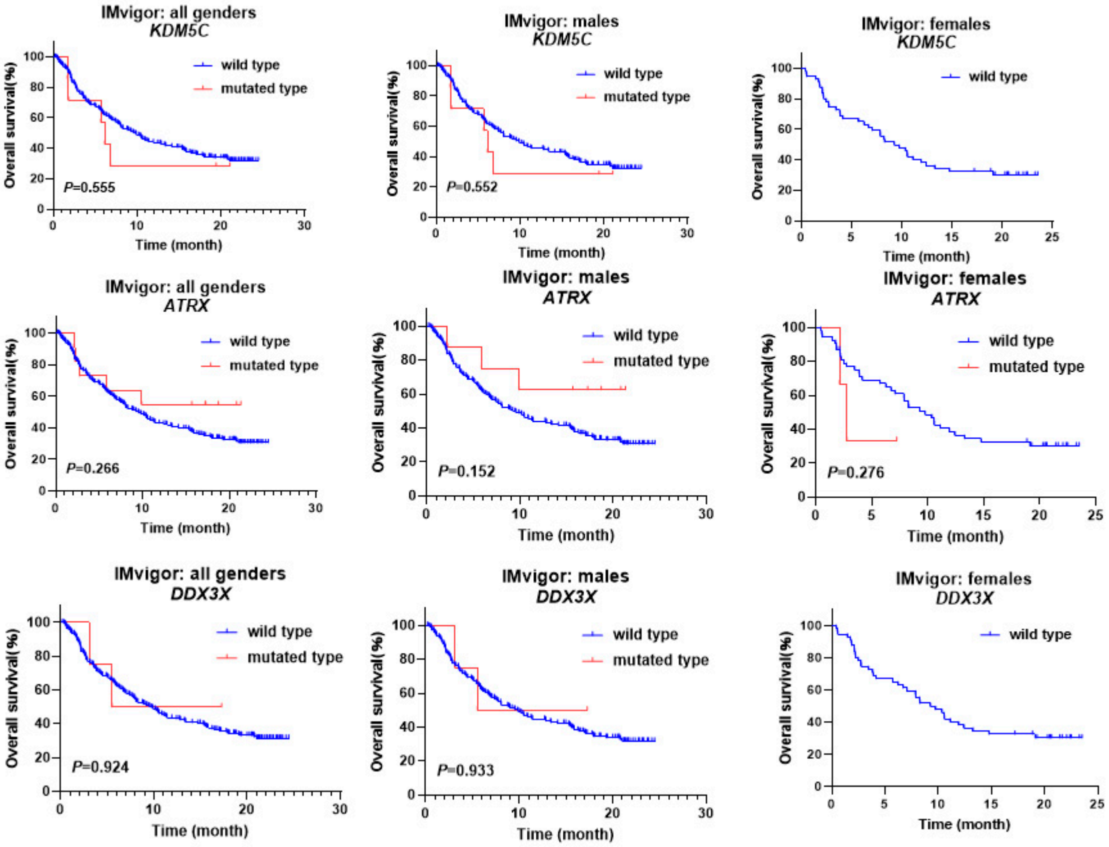

Figure S1. The gender-biased difference in overall survival according to mutation status of 3 genes (*ATRX, DDX3X, and KDM5C*) that escape from X chromosome inactivation in the IMvigor 210 study in all genders, males, and females.

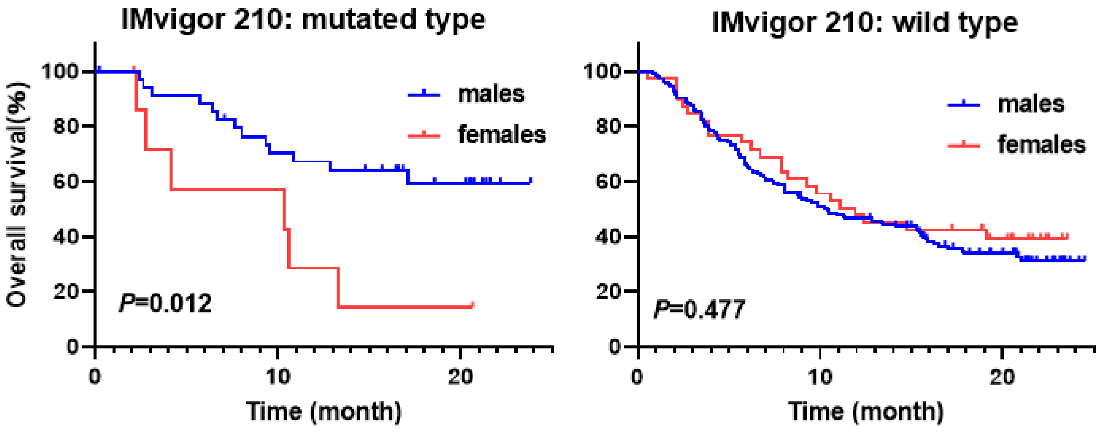

Figure S2. The gender-biased difference in overall survival according to *KDM6A* mutation status in the immune therapy cohorts (IMvigor 210).

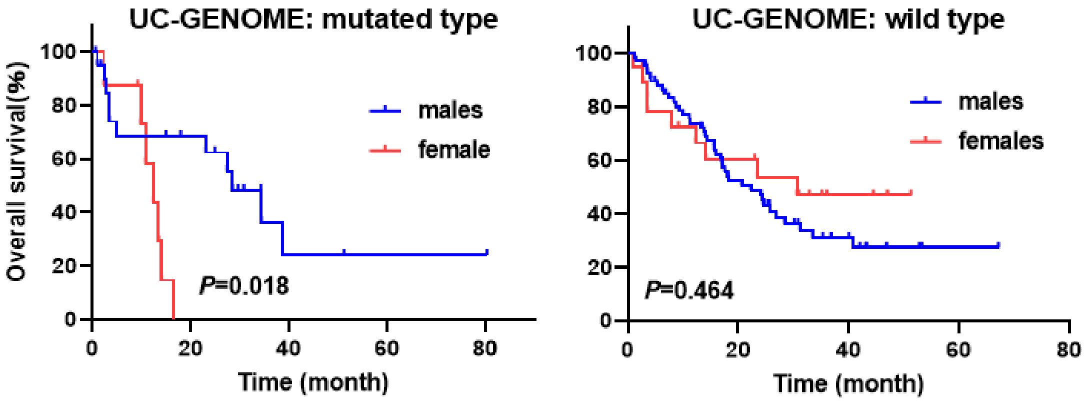

Figure S3. The gender-biased difference in overall survival according to *KDM6A* mutation status in the immune therapy cohorts (UC-GENOME).
